# Stakeholders’ perspectives and willingness to institutionalize linkages between the formal health system and informal healthcare providers in urban slums in southeast, Nigeria

**DOI:** 10.1186/s12913-022-08005-2

**Published:** 2022-04-30

**Authors:** Obinna Onwujekwe, Chinyere Mbachu, Victor Onyebueke, Pamela Ogbozor, Ifeyinwa Arize, Chinyere Okeke, Uche Ezenwaka, Tim Ensor

**Affiliations:** 1 Department of Pharmacology and Therapeutics, Health Policy Research Group, College of Medicine, University of Nigeria, Enugu-Campus, Enugu, Nigeria; 2 Department of Health Administration and Management, University of Nigeria, Enugu-Campus, Enugu, Nigeria; 3 Department of Community Medicine, College of Medicine, University of Nigeria, Enugu-Campus, Enugu, Nigeria; 4 Department of Urban and Regional Planning, University of Nigeria, Enugu-Campus, Enugu, Nigeria; 5 Department of Psychology, Enugu State University of Science and Technology, Enugu, Nigeria; 6 Nuffield Centre for International Health and Development, University of Leeds, Leeds, UK

**Keywords:** Institutionalization, Formalization, Integration, Linkages, Pluralistic health system, Nigeria

## Abstract

**Background:**

The widely available informal healthcare providers (IHPs) present opportunities to improve access to appropriate essential health services in underserved urban areas in many low- and middle-income countries (LMICs). However, they are not formally linked to the formal health system. This study was conducted to explore the perspectives of key stakeholders about institutionalizing linkages between the formal health systems and IHPs, as a strategy for improving access to appropriate healthcare services in Nigeria.

**Methods:**

Data was collected from key stakeholders in the formal and informal health systems, whose functions cover the major slums in Enugu and Onitsha cities in southeast Nigeria. Key informant interviews (n = 43) were conducted using semi-structured interview guides among representatives from the formal and informal health sectors. Interview transcripts were read severally, and using thematic content analysis, recurrent themes were identified and used for a narrative synthesis.

**Results:**

Although the dominant view among respondents is that formalization of linkages between IHPs and the formal health system will likely create synergy and quality improvement in health service delivery, anxieties and defensive pessimism were equally expressed. On the one hand, formal sector respondents are pessimistic about limited skills, poor quality of care, questionable recognition, and the enormous challenges of managing a pluralistic health system. Conversely, the informal sector pessimists expressed uncertainty about the outcomes of a government-led supervision and the potential negative impact on their practice. Some of the proposed strategies for institutionalizing linkages between the two health sub-systems include: sensitizing relevant policymakers and gatekeepers to the necessity of pluralistic healthcare; mapping and documenting of informal providers and respective service their areas for registration and accreditation, among others. Perceived threats to institutionalizing these linkages include: weak supervision and monitoring of informal providers by the State Ministry of Health due to lack of funds for logistics; poor data reporting and late referrals from informal providers; lack of referral feedback from formal to informal providers, among others.

**Conclusions:**

Opportunities and constraints to institutionalize linkages between the formal health system and IHPs exist in Nigeria. However, there is a need to design an inclusive system that ensures tolerance, dignity, and mutual learning for all stakeholders in the country and in other LMICs.

## Background

In many low- and middle- income countries (LMICs) including Nigeria, informal healthcare providers (IHPs) account for a significant proportion of health service delivery to underserved and vulnerable populations that are found in urban slums and rural areas [1, 2]. Furthermore, in countries like India and Bangladesh, IHPs provide a large proportion of rural outpatient services despite legal prohibition of their services (Bloom, et al., 2011; Gautham, et al., 2014). In Nigeria, evidence shows that IHPs such as patent medicine vendors (PMVs) and traditional birth attendants (TBAs) provide roughly 50% of all child services in Nigeria [3]. It has been found that PMVs are reported to be the first source of care (47%) for illnesses occurring among children under five years of age, and were found to be the preferred source of health care services for many urban poor [2, 3]. Furthermore, more than 50% of deliveries are assisted by an unskilled provider and TBAs account for 20% of the unskilled providers [4]. Many IHPs are important sources of healthcare in rural and urban slum areas and provide easy access to essential medicines and health services [5, 6]. With the poor coverage of health insurance [7], reliance on out-of-pocket payments for healthcare [8] coupled with uneven distribution of healthcare facilities and services in urban slums [9], there is an increasing reliance among the urban poor on the more accessible and affordable informal providers [10].

Urban slums have a reputation as global unhealthy and ultra-vulnerable places that pose the most stringent challenges for public health interventions. These unhealthy places retain heavy disease burdens, extraordinary infrastructural and socio-economic deprivations as was most evident during the Covid-19 lockdowns [11–13].

IHPs present opportunities to improve provision and access to essential health services in underserved and under-resourced areas such as urban slums, particularly in this era of people-centeredness [4]. Studies have shown that the informal providers operate more in the urban slums and rural areas, and are patronised largely by migrants and people of low socio-economic status [14]. The majority of IHPs are also known to deliver low quality services as evidenced by inadequate drug provision, poor adherence to clinical national guidelines due to ignorance and a lot of gaps in knowledge and practices of these informal providers [1, 14, 15].

Although a few IHPs have some level of informal training through apprenticeships, seminars, and workshops, they are typically not mandated by any formal institution. In most cases, IHPs are not registered with any government regulatory body and operate outside of the purview of official registration and regulations. Informal service providers may or may not have occupational associations. If such associations exist, they are primarily focused on networking and business activities, and minimal self-regulation is provided by these associations.

Studies have shown that PMVs and formal health facilities depend on one another in providing health services in urban, slum and rural areas [6, 16]. Despite their interdependencies, IHPs are not properly integrated or formally linked into the health system. For example, health service data from IHPs are not captured in the national health information system [14], and although clients may be referred from IHPs to public health facilities, and vice versa, these referrals are informal and unspecific in nature [6]. Although the contributions of informal healthcare providers are somewhat recognized in Nigeria’s health policies and strategic plans [4, 17–19], there are no clear mechanisms for integration into the National health system. Notwithstanding, recent reviews of Nigerian health system reforms continue to pay scant attention to informal-formal health service linkages or shun them altogether [8, 20]. In the absence of more consistent and structured linkages between informal and formal providers, the consequence of ‘fragmentation’ of health services and poor quality of care will be borne by service users, particularly women and children, many of whom are found in slums and poor urban neighborhoods. Hence, there is a critical need to strengthen and support more institutionalized linkages of the formal with in-formal health service providers so as to improve quality and continuity of care in underserved areas such as urban slums.

This paper provides new information on the feasibility of strengthening and institutionalizing linkages between the formal health systems and informal service providers in Nigeria. It is also necessary to create these linkages as the informal health providers are still preferred by many in the rural and urban slum communities due to convenience of proximity and ease of access, affordability, social and cultural preferences and conformity with their beliefs [2, 6, 15]. Policies of engagement are therefore needed to institutionalize these linkages and enforce mechanisms for compliance, especially where absence of a regulatory framework exists.

The paper highlights the existing relationships between the formal health system and informal service providers, stakeholders’ willingness to institutionalize existing and potential linkages, proposed strategies (opportunities) for bringing this to reality, and perceived threats to achieving success. It provides guidance to researchers, policy makers, programme managers, and professional bodies who are seeking to implement strategies for reinforcing and formalizing relationships between health service providers in the context of multiplicity of providers.

## Methods

### Study design and area

Qualitative research methods were used to elicit the views of key stakeholders on their perspectives and willingness to institutionalize linkages between the formal health system and informal healthcare providers in urban slums in Anambra and Enugu States, southeast Nigeria. The two states were purposively selected based on their contiguity and retention of large swathes of urban slums. Furthermore, Onitsha in Anambra State has among the largest open drug markets in Nigeria, a situation fueling fake and adulterated drugs alongside thriving informal health services [21]. The target slums and focus of the study are Okpoko slum area in Onitsha and Abakpa slum, the two largest urban slums in Anambra and Enugu State respectively.

**Fig. 1:**
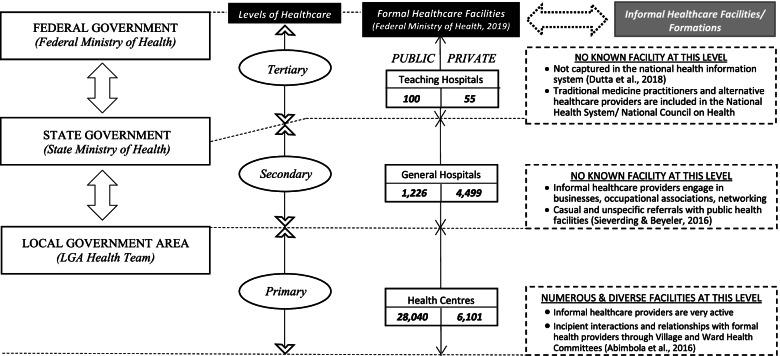
Structural dynamics of Nigeria’s health system and the subordinate place of formal-informal healthcare linkages

Anambra and Enugu States are located in the southeast geopolitical zone of Nigeria with total population respectively of 4.2 and 3.3 million, and each with an average annual growth rate of 2.8% [22]. The health system in both States, like the rest of the country, is organized in three tiers for service delivery – primary, secondary and tertiary subsystems. In each State, the Ministry of Health oversees and regulates the activities of health-care. Figure 1 highlights the structural dynamics of Nigeria’s health system and the subordinate place of formal-informal healthcare linkages.

### Study participants

The study adopted a stakeholder consultation approach [23]. The participants were purposively selected based on their roles and involvement in coordination and/or health service provision to ensure a representation of key stakeholders in the formal health system and IHPs, as well as to elicit diverse perspectives on the topic of study. The key informants were drawn from the State Ministry of Health, State Primary Health Care Development Agency, State Health Insurance Agency, LGA health authority, and Regulatory agencies. Others were drawn from professional associations of formal and informal service providers, and popular formal and IHPs in selected urban slums in the two States. In all, 43 respondents were selected, comprising 12 State level decision makers, five LGA health authority officials, eight representatives of regulatory bodies, 10 informal providers and eight formal providers (Table 1). All the participants were fully involved in the study and there was no dropouts. In the study, IHPs are defined as health providers who have not received formally recognized training with a defined curriculum from an institution (such as government, non-governmental organization, or academic institution), and include, but are not limited to, PMVs, TBAs, bone setters, traditional medicine practitioners and alternative health care providers [15].

**Table 1:** Participants interviewed and their categories in the formal and informal health systems in Anambra and Enugu States, Nigeria

Stakeholder category	No. of participants
**1. State Policy and decision makers** • State Primary Health Care Development Agency (Executive Secretary) • State Ministry of Health ◦ Permanent secretary ◦ Department of Pharmacy ◦ Department of Medical Services (providers licensing officer/desk) • State Health Insurance Agency (Executive Secretary)	10
**2. State programme managers** • Malaria or RMNCAH or HIV/TB or NCDs	2
**3. Local government health authority** (Enugu East, Enugu North, Enugu South, Onitsha North, Onitsha South LGAs): PHC coordinator (HOD of health)	5
**4. Regulatory bodies with chapters in Anambra & Enugu States** • Pharmaceutical Council of Nigeria • Nursing and Midwifery council • Community Health Practitioners’ Council • Traditional Medicine Board	8
**5. Informal health service providers** • State Chairman NAPPMED • Coordinator of PMVS or director (MD) of most popular PMV in each slum area • State Chairman Traditional Healers/medicine practitioners • Chairperson Traditional Birth Attendants • Chairperson Bone setter’s association	10
**6. Formal health service providers (in Abakpa and Okpoko)** • OIC of most popular PHC in the slum area • Medical director of most popular Community pharmacy in the slum area • National Medical Association • National Association of Nurses and Midwives	4
**7. Professional associations** • National Medical Association • National Association of Nurses and Midwives	4
**Total**	43

Source: Authors’ compilation, February 2021

### Data collection

Data were collected by experienced male and female health researchers who have been conversant with qualitative data collection using two different pretested key informant interview guides (KII) that were developed by the researchers for the purpose of the study. One KII guide was used for policy and decision makers, and the other KII guide was used for healthcare providers. The interview guides for KII elicited information on past and current linkages between the two groups, their perception on envisaged benefits and possible challenges of strengthening and formalizing such casual and fledging connections. Data was collected between February and March 2021 but prior to this, a stakeholder meeting was conducted with these respondents to establish a relationship with them and inform them of the upcoming interviews.

Most of the interviews were audio-recorded expect for two that were captured using handwritten notes because the respondents were not comfortable to be recorded. Each interview was conducted face-to-face, in their workplaces and lasted about 40–45 min. Prior to commencing, all participants were informed of the objectives of the study. Written consents were obtained from all participants having informed them of the purpose of the study, benefits and risks of participating in the study, and their rights to voluntary participation and confidentiality of data, as well as some background about the interviewer. Permission to audio-record interviews/discussions was also obtained. Data was collected till saturation was observed as respondents of corresponding fields or preoccupations were giving the same information. All interviews were transcribed and emerging responses were analysed thematically.

### Data analysis

Transcripts of audio-recorded interviews were compared with handwritten notes to generate a complete transcript. In synthesizing the data, findings from informal health sector respondents were analyzed separately from findings from formal health system respondents.

Each transcript was read and summarized by the interviewer. The findings from each interviewer were compared during a de-brief meeting, and recurrent/emerging themes were used to generate the final framework for narrative synthesis of findings. The themes were identified in advance and they include: i) characteristics of service providers in urban slums, ii) existing linkages between informal providers and the formal health system – types, stakeholders involved, what has worked well or not worked well; iii) stakeholders’ willingness to institutionalize existing (and potential) linkages – including reasons; iv) proposed strategies (opportunities) for institutionalizing linkages (including proposed stakeholders and their envisaged roles) and; v) challenges (potential threats) to institutionalizing linkages between informal providers and the formal health system.

## Results

### Existing linkages between the formal health system and informal healthcare providers

Both categories of respondents understood linkages to mean relationships or collaborations that exist between informal and formal service providers, with the aim to ensure that quality health services are provided to the population. Some formal sector respondents acknowledge that informal service providers do contribute to filling the gaps in access to health services for the underserved, poor and vulnerable populations:

*“It is imperative for State Ministry of Health policymakers to understand that this people [informal providers] play a very important role, and that for us to succeed in getting our health indices in the right direction, there must be a linkage between these two sectors” (State policy maker, Anambra).*

The types of linkages between the formal health system and informal providers which were identified by formal sector respondents as currently existing in the urban areas include: i) registration and standardization of practice of informal providers by the Ministry of Health, either directly or through one of its agencies, ii) training of informal health providers, iii) provision of free commodities by the government, usually through the Ministry of Health to the IHPs, iv) referral of clients to formal facilities, and v) data reporting from IHPs.

Respondents from the informal sector identified existing linkages with the formal health system that are similar to those mentioned by their formal sector counterparts, and these include: i) registration of IHPs, ii) trainings from formal sector actors, iii) referral of clients, and iv) data reporting to specific health programmes. Table 2 highlights the existing linkages identified by each category of respondents, and the numbers of respondents that mentioned these linkages.

**Table 2:** Linkages that exist between the formal health system and informal service providers in Anambra and Enugu States, Nigeria

Types of linkages	Identified by formal sector respondents	Identified by informal sector respondents
Registration of informal service providers and standardization of practice by the formal health system (including enlisting of traditional medicine unit into the State Ministry of Health)	6 +	1 +
Training of informal service providers by stakeholders in the formal health system	6 +	1 +
Referral of clients from informal service providers to formal service providers	6 +	1 +
Data reporting on specific programs from informal service providers to the formal health system	6 +	1 +
Provision of free commodities such as insecticide-treated bed nets, to informal service providers	6 +	0

Source: Authors’ compilation, *February* 202*1*

### Stakeholders’ willingness to institutionalize existing (and potential) linkages between the formal health system and IHPs

#### Perspectives of respondents in the formal health sector

Majority of the formal sector respondents are optimistic about formalizing the relationship between the formal health system and IHPs. They believed that it would be accepted by many, and has the potential to create a synergy and improve access to quality services for people.

The rationale behind this view includes the fact that informal healthcare providers could expand some health services, especially in underserved urban slums and rural communities, where there are either poorly equipped or no health facilities at all. Hence, if properly trained and supervised, they could contribute to filling the gaps in access to quality health care service for vulnerable populations. For examples, TBAs have participated in HIV case identification and linkage in care, and PMVs have been engaged to provide malaria testing services and treatment of uncomplicated malaria.

*“Some of the linkages have been found beneficial – for instance TBA’s participation in the HIV case identification and referrals and PMV’s participation in Malaria diagnosis and treatment. They could be easily trained in referrals as the people believe more in them than formal health providers* (State programme manager, Enugu)

*“These informal providers are reachable and accessible to the wider community already, so integration will help fill the gap of health care access issues”* (State policy maker, Enugu).

Another reason for their optimism about the proposed linkage is that incorporating informal healthcare providers into the formal health system will enable better regulation and/or monitoring of their practice and products. This could contribute to reduction in the sale of substandard (fake or expired) drugs to clients and the general public. As an official of a key regulatory agency, recalled:

*“Very willing! Our people are patronizing them (traditional medicine dealers) and there is no guideline for controlling their activities. In fact, it is better to institutionalize linkages so that our people will have quality care”* (Regulatory agency, Anambra).

The final reason for the positive outlook of a number of formal sector respondents is that traditional medicine practice is recognized all over the world for its usefulness in patient care [24, 25]. Moreover, in some Asian countries, it has been successfully integrated into the formal health system. To further buttress this, a respondent stated that,

*“In China, both the orthodox and un-orthodox medicine are well recognized, and they work hand-in-hand. Their acupuncture and local herbs are still used, and they work till date”* (State Policy maker, Enugu)*.*

*“I think the formal sector is willing because nobody can deny the fact that our people are patronizing the informal providers. The government can help by making policies and giving it a legal bite”* (State Policy maker, Anambra).

However, some formal sector respondents are averse to any formalization of linkages between the formal health system and informal healthcare providers. Some reasons for this pessimism include what they consider to be the impracticability of such a venture owing to limited or lack of training of informal providers, poor quality of care, lack of global recognition of the IHPs, and the fact that the formal health system is not equipped to manage the integration of informal healthcare providers.

Some of the respondents’ remarks are notable:

*“No institution in the world has recognized Traditional Birth Attendants, so Nigeria should not be the first. So, they shouldn’t be used as alternatives in any place”* (State policy maker, Enugu).

*“Quality is the underlined word, at an affordable rate but the functional words are, access, quality, affordability. PMVs cannot give all of these to patients, especially quality”* (State policy maker, Anambra).

*“For now, I’m not willing to have close institutional links with the informal providers. What will improve provision of appropriate health services is employing more health workers in the PHCs and motivating them. If the government does that, people will start patronizing PHCs instead of informal providers”* (Formal health service provider, Anambra).

#### Perspectives of respondents in the informal health sector

From the perspective of the informal sector respondents, the majority expressed a willingness to have institutionalized links with the formal health system. They were of the opinion that formalizing already existing and new relationships will help informal healthcare providers in various ways.

They were optimistic that it would grant them opportunities to attend training and receive supportive supervision from the government. These would contribute to improving their skills and the quality of care they provide to clients. Consequently, they will gain more recognition in the community, which could translate into increased patronage, more clientele and more money.

Secondly, it would help the informal healthcare providers to identify and coordinate their members better, also to forestall the proliferation of unregistered informal providers, including those who infiltrate from outside the community.

Finally, it would contribute to regulating the practice of informal providers thereby reducing quackery and malpractice.

*“We are willing to have close institutionalized links with the State Ministry of Health. We believe that will give us more opportunity to showcase our products. We want to be recognized by the government so that people can boldly use us as an alternative to hospitals”* (Informal health service provider, Anambra).

*“That is what we are praying for. We are willing to have close links with the Ministry of Health. However, we don’t want to work with the formal providers. We want every group to have the opportunity to present what they have. Government should build treatment centres for us, employ us and supervise our activities”* (Informal health service provider, Anambra).

However, a considerable number of respondents from the informal health sector highlighted that informal healthcare providers such as PMVs may not want any linkage beyond the current relationship they have with the formal health system. This is because they are uncertain about the outcomes of a government-led supervision and fear that it could impact negatively on their practice. Some concerns expressed by the informal sectors respondents about how the integration/linkage could affect their practice, include,

- Loss of clientele: informal providers do not want anything that will make them lose their clients or threaten their practice.
- Increased taxation: they fear that they will be taxed more in the course of institutionalizing these linkages, complaining that they do not make much money as it stands
- Disparagement by formal health system: informal providers fear that they will be addressed as ‘quacks’ by formal service providers
- Limitation of practice: they also fear that their scope of work may be forcefully limited.

### Proposed strategies for institutionalizing linkages between formal and informal providers

Respondents from the formal and informal sectors proposed various strategies that could be used to formalize linkages between informal service providers and formal services providers in the urban slums. There were some overlaps in the suggestions made by both categories of respondents (Table 3). However, there was the tendency for formal sector respondents to focus their recommendations on strategies that will enhance regulation and control of the informal healthcare providers.

**Table 3:** Proposed strategies for institutionalizing linkages between the formal health system and informal health providers

Proposed strategies	Identified by formal sector respondents	Identified by informal sector respondents
Advocacy to policymakers and gatekeepers	Seminars and sensitization workshops on integration, continuous stakeholders’ engagement	Open discussions on integration to ensure transparency
Mapping of informal providers in urban slum areas	Formalization of linkages with informal service providers involves obtaining information on who informal providers are, where and how they are operating	Mapping and categorization of informal providers in urban slums (e.g. PMVs, TBAs, TMPs, Bone setters, etc.) to help regulate their practices and control profusion of quacks questionable remedies.
Registration and accreditation	Creation of a legitimate association whose functions includes registration, renewal of licenses and accreditation	Creation of a legitimate board whose functions includes registration, renewal, accreditation, and discipline of defaulters
Training and certification	Training of informal providers on preventive care, data collection and client referrals. Encouraged certified training programmes for informal service providers	Training and re-training of informal service providers on rapid diagnostic tests, drug resistance, midwifery, early warning signs and emergency care, record keeping and infection prevention. Training should be based on curriculum and certified for only registered members
Engagement in service delivery	Involving the informal providers in curative care in very remote areas where there are no functional primary health centers. Standard referral protocols are encouraged	Advocates for more official recognition and support as contributors to health service delivery. (e.g. creation of unit in the Ministry of Health to coordinate and provide link with Informal sector, formal employment as a cadre of health workers in public service, a government-sponsored hospital complex for informal health providers)
Monitoring and supportive supervision	Activities of informal providers should be properly monitored and supervised by the Ministry of Health and other relevant agencies	Expressed preference for supportive supervision devoid of criticisms, condemnation or disparagement by Ministry of Health and relevant agencies
Platforms for communication	Monthly/quarterly meetings to facilitate referrals from informal providers to the formal providers. Monthly meetings were encouraged	Establish communication and open discussion channels between the formal and informal sectors for knowledge and idea sharing

Respondents noted that standard referral protocols should be introduced, with functional feedback mechanisms.

The informal health sector respondents (specifically the traditional medicine practitioners) emphasized that they would want more recognition by the formal health system as contributors to health service delivery. Recognition in the forms of creating a desk/unit for them at the State and the LGAs where they reside so they are closer to the community, and formal employment by the government as a cadre of health workers. They also propose the establishment of a government-owned traditional medicine treatment facility.

The proposed strategies are subsequently presented for both categories of respondents, with clear distinctions of perspectives where necessary.

#### Advocacy to policymakers and gatekeepers

The formal sector respondents proposed that targeted advocacy to policymakers and gate keepers in the formal health system could result in appreciation of the potential contributions of informal service providers to the health system. Secondly, it would pave the way (generate ‘political’ support) for other proposed strategies for creating linkages between formal and informal providers. Some respondents suggested advocacy tools such as:

*“Seminars and sensitization workshops on the importance of integration of formal and informal health providers”* (Regulatory agency, Enugu)

*“Continuous stakeholder engagement on linkages and support (empowerment) issues to ease grey areas”* (State policy maker, Enugu).)

Informal sector respondents also supported the need for advocacy. They opined that seminars and sensitization meetings will enable transparency and ensure that every party feels included.

*“Here, a forum for discussion of the agenda for integration will be created, so that everybody will understand the plan and see that there are no hidden agenda”* (Informal health service provider, Enugu)

#### Mapping of all informal providers in urban slums

Stakeholders in the formal sector were of the opinion that a primary step in the formalization of linkages with informal service providers it is to collect information on who these informal providers are, where they operate, and how they operate. Hence, a detailed mapping of all informal service providers.

Several formal sector respondents in both states noted that there is a need to first of all locate these service providers to know exactly what they are doing, how they acquired their knowledge/skills, and what levels of education/knowledge/skills they possess.

*“First, locate them and know exactly what they are doing”* (Formal health service provider, Anambra).

#### Registration and periodic accreditation of informal healthcare providers by a recognized board

The formal sector respondents suggested that in order to ensure that standards of care are maintained in the proposed linkage between formal and informal healthcare providers, there is a need to first of all create a recognized (legalized) board of association. This board will then be responsible for accrediting informal providers (based on a set of standards), registering these providers, and renewing practicing licences following yearly re-accreditation of providers. It was envisaged that this process will ensure that the practices of informal providers are regulated, and they are accountable.

A supporting quote from a policymaker reads, *“Enforce registration with an Association. This will ensure accountability of their members, identification and availability of data on their activities in the community”* (State policy maker, Enugu)*.*

Informal sector respondents also appeared to affirm the views of formal sector respondents about regulating the practice of informal healthcare providers. In addition, they proposed that penalties such as closure of premises should be enforced for malpractice, non-adherence to clinical guidelines, and practicing with expired licenses (annual registration).

#### Training and certification of informal health providers

The need for training and certification of informal providers was reiterated by formal and informal sector respondents alike. In the opinion of the formal sector respondents, training informal providers on how to provide preventive care will contribute to sustaining any linkage with formal providers. The training will also encompass data collection and client referrals.

*“Enforce certificate courses for registered Informal health providers through accredited health schools”* (State policy maker, Enugu)

“[informal providers should] *Receive regular certified training schemes and retraining of their members by the formal health workers to upgrade their capacity to identify early warning signs for referral* (Informal health service provider, Enugu).

It was suggested that such training schemes should be standardized (using training manuals or frameworks or curriculum), organized by the formal health system or accredited schools of health, and certified (that is, certificates should be provided to participants) to motivate the informal providers. And only registered informal providers should benefit from such guidance and capacity-building schemes.

Formal sector respondents anticipate that since previous trainings organized by the Ministry of Health for informal providers have worked well in building their capacity, introducing the strategy in a formalized linkage will contribute to improvements in quality-of-service delivery to clients/communities served.

*“Government can create a framework for training […] to ensure the informal providers do not go beyond their scope. For instance, PMVs should only sell over the counter drugs”* (Professional association, Anambra)

*“Trainings* [should be] *guided by a training manual to help informal providers in areas of health sensitization, referral and reporting of community incidence”* (State policy maker, Enugu)

There was also the view that the private education sector should contribute by establishing training centers in rural areas using, *“curriculum tailored to provide short term (2-year certifications) for informal health providers”* (State policy maker, Enugu).

Informal sector respondents highlighted the important areas of training for informal service providers to include, how to conduct rapid diagnostic tests for malaria and HIV, and issues of drug resistance for the PMVs. For the TBAs, training should encompass modern midwifery skills, early warning signs and emergency care, prompt referral, infection prevention and control, and record keeping.

Informal sector respondents also affirmed that issuing certificates of training to informal healthcare providers incentivizes them greatly. It was stated that.

*“The certificates are very important to them* [informal providers] *as they exhibit it in their treatment rooms with the belief that it increases their client’s trust in them and attracts more clients as well”.* (State policy maker, Enugu).

#### Engaging informal healthcare providers in service delivery

Some formal sector respondents suggested that involving informal providers in preventive or curative care for common health problems could be used to link them into the formal health system. However, it was clearly stated that involving them in curative care should be an exception rather than the norm. For instance, in very remote areas where functional primary health centers or community pharmacies do not exist, patent medicine vendors can be contracted by the public sector to dispense over-the-counter medications for minor illnesses.

One respondent emphasized that alternative (traditional) medicine practitioners could be formally engaged to provide curative care to clients who choose unorthodox medicine. She however noted that treatment procedures and medicine prescriptions must be standardized using scientific specifications.

Irrespective of the level of engagement of informal providers in service delivery, referral of clients was emphasized. Respondents noted that standard referral protocols should be introduced, with functional feedback mechanisms.

The informal sector respondents (specifically the traditional medicine practitioners) emphasized that IHPs would want more recognition by the formal health system as contributors to health service delivery. Recognition in the forms of creating a desk/unit for them at the State and the LGAs where they reside so they are closer to the community, and formal employment by the government as a cadre of health workers. They also propose the establishment of public-owned traditional medicine treatment centers.

#### Monitoring and supportive supervision

Monitoring and supervision of informal healthcare providers was a recurrent recommendation of both categories of respondents, albeit with some variations in the level and scope of the supervision.

Formal sector respondents strongly felt that the activities of IHPs should be properly monitored and supervised by the Ministry of Health. An official of the Nigerian Medical Association stated the organization’s willingness to support the formal health system in identifying quack practitioners, with the help of an anti-quackery unit. However, he worried that for capacity and funding limitations, both the formal sector and the informal sector are not yet prepared to handle any form of integration or linkage of service providers.

*Regulation to curb quackery in health care is very important. There are some registered hospitals that are not owned and managed by a doctor. The government should fight quackery. There is no funding for effective supervision and monitoring. So many things need to be corrected in the formal sector as it is now and informal sector before we can think of integration* (State policy maker, Enugu)

Another formal sector respondent suggested the use of key performance indicators in monitoring the activities of informal providers. He said that, *“The best way is to have clearly stated key performance indicators (KPIs), you state what you want to achieve with targets and then collect data about this your KPIs and measure them, whatever is not measured is not done. So, my opinion would be to get key performance indicators, get target and then measure this over time like having indicators like how many first-time mothers came to TBAs, how many were referred *etc.,* which will be helpful to monitor practice* (State policy maker, Anambra).

The rationale for the proposed strategy among the formal sector respondents is that monitoring and supportive supervision of informal providers will engender accountability and motivation to do what is right.

On the other hand, the informal sector respondents expressed unease about IHPs being supervised by the formal health system (Ministry of Health), because it will limit the scope of their practice, and consequently their income/earnings. They stated a preference for supportive supervision that is without criticisms, condemnation or disparagement. They also proposed that the supervision should be done by government officials, professional associations and/or regulatory bodies, rather than by the formal providers. There was also the suggestion to integrate the supervision with the current regulatory visits by the Pharmaceutical Council of Nigeria which involves inspection of premises, enforcement of registration and regulation of practice to ensure adherence to guidelines. However, they emphasized that the financial burden of logistics for carrying out supervisory visits should be borne by the supervising agency and not the informal providers. They lamented that the reverse had been the case in the past, and PMVs have been made to bear the financial cost of being visited by the Pharmaceutical Council of Nigeria for inspection and renewal of practicing license.

#### Establish platforms for communication

Communication was highlighted as an important thread that will hold the fabrics of the proposed linkages between formal and informal providers. It was also opined that functional communication channels would facilitate referrals from informal providers to the formal providers. Respondents suggested:

*“Creating a forum where the formal and informal health providers can meet and discuss cordially as was done during the PATHS 2 project”* (Informal health service provider, Anambra)

*“There should be regular monthly/quarterly meetings involving their representatives of the informal health providers to deliberate on any emerging issues and ensure adherence to policies and laws are strictly done”.* (State policy maker, Anambra)

In addition, one of the respondents noted that *“Religious and traditional leaders should be involved because culture and religion made people to prefer bone setter and TBAs to orthopedic and maternity hospitals”* (State policy maker, Anambra).

### Proposed stakeholders and their contributions to institutionalizing linkages between the formal health system and informal healthcare providers

Respondents perceived that in order to make the process more effective, a multi-sector and multidisciplinary approach should be adopted for institutionalizing linkages between informal providers and the formal health system. It was suggested that the Ministry of Health should collaborate with other actors in the formal health system such as health professional associations and regulatory agencies, as well as non-health sectors such as ministries of women affairs, education and environment. Some of these ancillary ministries could also contribute to enforcing regulations.

Respondents proposed specific stakeholders and the roles they could play, and these are highlighted in Table 4.

**Table 4:** Proposed stakeholders and their roles in institutionalizing linkages between the formal health system and informal service providers

Stakeholders	Roles proposed by formal sector respondents	Roles proposed by informal sector respondents
Federal government	-	Soft loans to TMP to produce their products in large quantities for exportation. Send federal agencies to support them with trainings on product preservation and exportation processes
State government	Build training schools for traditional medicine	Build training schools for traditional medicine. Organize fairs for TMPs to showcase their products. Integrate informal providers’ services Provide ambulance at strategic locations to enhance referral especially in urban slums and rural communities
Local government	Establish an agency/desk to monitor and supervise informal service providers	
Ministry of Health and SPHCDA	Establish an agency/division to coordinate trainings, supervision and regulation of informal providers Registration and accreditation of informal providers and enforcement of standards of operation	Registration and accreditation of informal providers and enforcement of standards of operation Advocacy and sensitization on need for linkage between formal health system and informal providers
SPHCDA	-	Administration and evaluation of capacity building and possible funding
Pharmaceutical Council of Nigeria	Regulation of practice of PMVs, including development and enforcement of standards of operation and other guidelines Standardizing and regulating locally produced drugs and herbs	Regulation of practice of PMVs, including development and enforcement of standards of operation and other guidelines
Medical and Dental Council of Nigeria	Training on best practices in preventive care for all informal providers	-
Nursing and Midwifery Council of Nigeria	Training TBAs on best practices including cord care, sterilization of delivery and circumcision instruments, recognition of danger/warning signs, prompt referral Regulation of the practice of TBAs	
Association of General Private Medical Practitioners of Nigeria	-	Support integration efforts
Health development partners	-	Funding support – to implement proposed interventions Technical support – training of service providers
NGOs and CSOs	-	Engender community participation. Sensitization and advocacy
Health officers	-	Engender community participation
Informal health service providers	Prompt referral of cases to formal health services Adherence to recommended guidelines Mobilization of members to comply with registrations and accreditations	

### Perceived challenges to institutionalizing linkages between formal and informal providers

Formal and informal sector respondents were asked to reflect on what has worked well and not worked well in existing or previous relationships between informal healthcare providers and the formal health system.

From the perspective of formal health sector respondents, although the various trainings have been provided to IHPs, many maternity homes run by retired nurses are unregistered and therefore not supervised by the Ministry of Health. Furthermore, supervision and monitoring of informal providers by the Ministry of Health is hindered by lack of funds for logistics. Respondents also highlighted that data reporting and referrals from informal providers have been suboptimal. It has been impossible to get all the informal providers to submit their monthly data, and some informal providers do not refer clients till the case is advanced, and almost too late. Majority of informal providers have little or no education, job experience and either no or very little training thereby limiting their ability to provide appropriate healthcare services.

From the perspective of respondents in the informal health sector, it appears that most existing linkages have not worked in their favor. They viewed the referral system to be ineffective because there is no feedback from formal providers to informal providers who refer their clients. Training of informal providers has been infrequent (and almost non-existent) and their registration and regulation is undermined by bribery of officials of the regulatory agencies. They also fear that institutionalizing linkages will adversely limit their scope of their practice.

## Discussion

The findings show that in both study areas, there is the general willingness to institutionalize linkages between the formal health system and informal healthcare providers for diverse reasons. The linkage could encompass a number of aspects, including formal registration and issuance of license to practice based on certain criteria of IHPs, regulation of their activities and services provided to ensure that they are of good quality, supportive supervision for better service delivery, training to upgrade their knowledge, monitoring. The willingness of stakeholders in the formal health system to be formally linked with the informal healthcare providers stemmed from limitations of population coverage with health services, and past experiences of working with informal providers in the prevention of HIV and Malaria through early diagnosis and prompt referrals. The findings collaborate findings in other countries where informal healthcare providers are gradually being recognized as beneficial to the health system [3, 23], Moreover, national health policies have been developed that call for the integration of IHPs into national health systems [3].

Some respondents felt that the costs of institutionalizing the IHPs into the formal system may outweigh the benefits due to several factors such as unrealistic competition, false claims, sales of concoctions, extortion of citizens, and other unethical practices that pose grave dangers to public health and safety. On the contrary, informal providers crave for cooperation and linkages in order to ensure government recognition, legitimization and public acceptance. Another reason is that these alternative health providers feel left out (and even disrespected) by government and other formal health decision makers in policy formulation and development of health sector strategy.

Although respondents in the formal health sector have expressed the view that IHPs may be difficult to control and coordinate, evidence suggests that most informal providers understand the need and value for skilled health care. A study in Nigeria found that TBAs understood the implications of referrals and considered postnatal referrals for women after childbirth to be a means of boosting their reputation as competent care providers, attracting new clientele [26]. The informal providers have been shown to successfully participate in pilot studies that explored the utilization of different types of healthcare providers and factors associated with provider choice by insurance status in rural Nigeria where the informal providers made up respectively 73% and 78% of all consultations among insured and uninsured cases [27].

The more inclusive and better articulated views on the linkage opportunities expressed by State-level policy and decision makers seems to give way to rivalries and more combative views from professional associations. For example, the strong imperative to curtail the activities of quacks within informal providers. These findings are in line with the evidence that professional animosities exist between the formal and informal health providers and are seen more with the formal health workers in the private sector [6], whereas in other cases, formal health providers have good relationship with the informal health providers and provide informal advice to enable them practice efficiently [24]. In institutionalizing these linkages, formal health providers will therefore need to be persuaded that those informal providers can be trained and adequately regulated so that potential disadvantages of greater involvement, such as inappropriate treatment and delayed referral to formal providers can be mitigated.

Since idea of linkages is often selective (i.e., conceived with familiar informal providers in mind) an understanding of the diverse determinants and opportunities for the close linkage of the informal providers to the formal health system should, therefore, include, (i) mapping and personnel audits of formal and informal providers in order to locate them and know exactly what they are doing; (ii) regular training and retraining of informal providers to ensure quality of care; (iii) licensing, regular supportive supervision and regulatory oversight by relevant agencies, ministries and parastatals using clearly stated key performance indicators; (iv) creation of liaison offices or units, and appointment of representatives in the State Ministry of Health and other relevant AMPs for better enlightenment and to ward off inherent distrusts; (v) instituting open fora and mutual learning for exchange of ideas, where the formal and informal healthcare providers can meet and discuss cordially.

Of all the opportunities for closely linking the formal health system and informal healthcare providers identified above, most studies have shown the importance of training and capacity building [13, 14]. However, despite capacity building interventions, IHPs have been found to prescribe harmful and sometimes unnecessary medications [2]. Hence, any attempts to even legitimize the practice of IHP should incorporate a combination of strategies that will ensure compliance to standard operating procedures including reinforcements with printed media, participatory problem solving, referral systems, and supportive supervision [25].

Respondents from the informal health sector rightly expressed fears about the financial implications of their close linkage and integration into the formal health system, because it is known that institutionalizing and maintaining linkages entails investments in governance and leadership; and logistics for training and monitoring IHPs [28]. In order to encourage co-operation of informal providers, the government agency for supervision must be known and integrated with the supervising professional body to avoid duplication of supervisory visits that may be a financial burden to the government and informal system. Furthermore, establishing and maintaining linkages that has a robust framework are key to achieving systems linkages that are accountable, transparent and good means of disseminating good practices. Availability of effective regulatory mechanisms is essential to help shape the linkages. If the institutionalizing and maintenance of the linkage is not properly integrated, it may lead to many barriers between the formal and informal health systems, including: unsustainable funding; poor quality training; undefined roles between informal and formal careers; and poor communication between stakeholders and others [28].

Limitations of the study include the fact that the spread of participants may not have sufficiently represented all relevant stakeholders in the formal and informal health system, Secondly, all stakeholders interviewed were from one region in Nigeria, and stakeholders’ views may vary across the different regions.

## Conclusions

Opportunities exist to strengthen and institutionalize linkages between the formal health system and informal healthcare providers. Since informal healthcare providers have a massive clientele, particularly in the rural areas and urban slums, related policy formulation and measured legalization of their practices are imperative for an integral health system. Integration of informal providers to the formal health system may re-orient local health systems for improved service delivery through a people-centered approach, thereby expanding and deepening access to quality health care for clients. However, the proposed institutionalization has to be both inclusive and transparent, and implemented in such a manner as to ensure tolerance, dignity and respect, as well as mutual learning. Once designed and instituted, the management of such a proposed (expanded) health system will require multidisciplinary and multi-sector approaches.

Finally, besides requiring continuous broad-based implementation research on the actual integration of formal and informal providers, factors such as government recognition, trust-building and mutual respect among all healthcare providers as well as public awareness and acceptance could promote linkages, and boost clientele for informal providers. Government recognition could contribute to authenticating informal providers as alternative entry points into the formal health system. It will also aid their coordination and supervision in order to reduce infiltration of fakes and quacks.

## Data Availability

The datasets used and/or analysed during the current study are available from the corresponding author on reasonable request.

## Abbreviations

IHP: Informal health provider
KII: Key informant interview
LGA: Local government area
PHC: Primary health centre
PMV: Patent medicine vendor
TBA: Traditional birth attendant
KPI: Key Performance Indicator
